# Impact of Safety Device Features Upon Motor Vehicle Collision Facial Fracture Traumas: A Five-Year Retrospective Study in the Appalachian Tri-State Area

**DOI:** 10.7759/cureus.61659

**Published:** 2024-06-04

**Authors:** Armein Rahimpour, Jacy Baxter, David A Denning, Peter Ray, Barry Rahman

**Affiliations:** 1 General Surgery, Marshall University Joan C. Edwards School of Medicine, Huntington, USA; 2 Plastic and Reconstructive Surgery, Marshall University Joan C. Edwards School of Medicine, Huntington, USA

**Keywords:** automotive safety, appalachian region, safety devices, motor vehicle crash (mvc), facial fractures

## Abstract

Motor vehicle collisions (MVCs) represent a significant public health concern, contributing to substantial morbidity and mortality globally. Despite advancements in vehicle safety technology, the impact of safety measures on facial fractures in MVCs remains poorly understood, especially in regions with diverse road conditions like the Appalachian tri-state area. This retrospective study analyzed data from two regional level II trauma centers in Appalachia, focusing on MVC-related facial fractures and safety device usage from January 2017 to December 2021. Descriptive statistics and logistic regression models were employed to assess associations between safety devices and injury outcomes. The study comprised 85 participants, categorized into groups based on safety device usage. Results indicated no significant differences in demographic characteristics, injury severity, surgical intervention rates, or specific facial fractures among groups. These findings challenge some previous research suggesting the protective effects of safety devices on facial fractures in MVCs, underscoring the complex nature of injury prevention in this context. While limitations such as retrospective data collection and sample size constrain generalizability, this study contributes valuable insights for informing injury prevention strategies and trauma care practices in Appalachia and beyond.

## Introduction

Motor vehicle collisions (MVCs) represent a pervasive public health concern, inflicting a substantial burden of morbidity and mortality worldwide. According to the National Highway Traffic Safety Administration (NHTSA), MVCs claimed the lives of 38,680 individuals in the United States alone in 2020, underscoring the significant impact of these accidents on society [1]. Moreover, the Centers for Disease Control and Prevention (CDC) identifies MVCs as the leading cause of death among individuals aged one to 24 years and the second leading cause among those aged 25-44 years [2]. Beyond fatalities, MVCs contribute substantially to non-fatal injuries, hospitalizations, and emergency department visits, imposing profound physical, emotional, and economic burdens on affected individuals and communities [3-4].

Despite ongoing efforts to enhance vehicle safety through technological advancements including airbags, seat belts, and crash avoidance systems, the influence of these safety measures on the occurrence and severity of facial fractures in MVCs remains poorly understood [5-6]. This knowledge gap is particularly pronounced in regions characterized by diverse road conditions and traffic patterns, such as the Appalachian tri-state area [7].

The Appalachian region presents a unique context for studying MVC-related injuries and safety interventions. With its rugged terrain, winding roads, and challenging driving conditions, Appalachia poses distinct challenges for motorists and healthcare providers alike. Moreover, socioeconomic factors including limited access to healthcare and disparities in injury prevention resources further magnify the complexity of MVC-related injuries in this region [8-9].

Previous research has highlighted the potential protective effects of safety devices, such as seat belts and airbags, in mitigating injuries sustained in MVCs. For instance, Cummings et al. demonstrated a reduced risk of facial fractures associated with seat belt usage among MVC patients [10]. Similarly, Haider et al. found that airbag deployment was correlated with a lower likelihood of severe facial injuries [11]. However, the generalizability of these findings to diverse populations and geographic settings, particularly within Appalachia, remains unclear [12].

This study seeks to address this gap in knowledge by conducting a retrospective review of MVC-related facial fractures among patients admitted to two regional level II trauma centers in the Appalachian tri-state area. By examining the association between safety device usage and the occurrence and severity of facial fractures, this study aims to provide valuable insights into the effectiveness of safety measures in preventing facial trauma in MVCs within the context of Appalachia. Furthermore, by contextualizing our findings within the broader literature on MVC-related injuries and safety interventions, we aim to inform evidence-based strategies for injury prevention and management in trauma care settings, particularly in regions with similar geographic and socio-economic characteristics [8].

The importance of this study lies in its potential to elucidate the role of safety devices in preventing facial fractures in MVCs within the unique context of Appalachia, thereby informing the development of targeted interventions aimed at reducing the burden of MVC-related injuries on individuals and society in this region. Moreover, by leveraging data from multiple trauma centers in Appalachia, this study offers valuable insights into the impact of regional variations in road conditions, traffic patterns, and healthcare resources on injury outcomes in MVCs. Ultimately, the findings of this study have the potential to inform policy decisions, guide public health initiatives, and improve the overall quality of trauma care for MVC patients in Appalachia and beyond [13].

## Materials and methods

The study was authorized by the Marshall University Institutional Review Board (IRB No. 1991703-3). In order to complete this retrospective study, patient records maintained in our trauma registry at Cabell Huntington Hospital and Saint Mary's Medical Center were reviewed. Both hospitals are academic teaching hospitals, regional referral centers, and American College of Surgeons-verified level-2 trauma centers in Huntington, WV.

The analyzed medical files belonged to patients who presented between January 1, 2017 and December 31, 2021. The Information Technology (IT) departments at each center were contacted to obtain data. The request included any patients above the age of 18 years who sustained a facial fracture secondary to an MVC and presented to the emergency department at the aforementioned trauma centers during the five-year period. In addition, we requested the age, gender, Injury Severity Score (ISS), and length of hospital stay from the IT team. The initial sample consisted of 616 patients. The collected data were centralized using Microsoft Excel software. The medical records for those MVC patients meeting the previously mentioned criteria were reviewed. Only those with clear documentation of both airbag deployment and seatbelt usage status were included in our analysis, accounting for the final sample size of 85 participants. The following data were extracted from the medical records: type of facial fracture (nasal, bilateral nasal, orbital, bilateral orbital, mandibular, bilateral mandibular, maxillary, bilateral maxillary, zygomatic, and bilateral zygomatic) and facial fractures requiring operation.

Descriptive statistics were used to summarize the sample characteristics. Continuous variables were reported as means ± standard deviations (±SD), median, first quartile, and third quartile. Categorical variables were reported as numbers (n) and percentages (%). One-way analysis of variance (ANOVA) was used to assess the age difference among the groups. The Kruskal-Wallis test was used for non-normally distributed continuous variables (i.e., hospital length). The chi-square test was used to determine if there were significant differences between groups based on seatbelt and airbag status for each categorical variable. Fisher’s exact test was used instead if the expected count was less than 5. Logistic regression models were performed to assess the association between safety devices and outcomes, using patients who used both seatbelts and airbags as the reference group. This model was used to determine the odds ratios and 95% confidence intervals for each predictor variable. All statistical analyses were performed by using SAS (SAS 9.4, SAS Institute Inc, Cary, NC, USA). Statistical significance was defined by the two-sided test with a p-value <0.05.

## Results

This study encompassed 85 participants categorized into four groups by safety device utilization. More than one-third of the participants used both seatbelts and airbags (n = 28, 32.9%), and 28 participants used airbags only (n = 32.9%). Twenty participants used seatbelts only, and nine participants did not use seatbelts or airbags. The mean age of the participants was 40 years (SD = 17), overall ranging from 18 to 85 years of age. More than half of them were male (n = 53, 62%), and 38% (n = 32) were female. The mean Injury Severity Score (ISS) was 14 (SD = 10), and the mean length of hospital stay was nine days (SD = 13). The distribution of participants across these groups did not significantly differ by gender (p = 0.46), age (p = 0.82), ISS (p = 0.25), or hospital length (p = 0.67) (Table 1).

**Table 1:** Sample characteristics by seatbelt and airbag status Data presented as n (%), excluding SD. n, number; %, percentage; SD, standard deviation; IQR, interquartile range.

Variables	Overall (n = 85)	Seatbelt and airbag (n = 28)	Seatbelt (n = 20)	Airbag (n = 28)	None (n = 9)	p-value
Age						0.82
Mean±SD	40±17	39±15	43±18	41±18	38±14	
Median (IQR)	39 (26, 50)	37 (29, 49)	48 (25, 56)	35 (27, 52)	39 (26, 48)	
Gender						0.45
Female	32 (38)	10 (36)	10 (50)	8 (29)	4 (44)	
Male	53 (62)	18 (64)	10 (50)	20 (71)	5 (56)	
ISS						0.25
Mean±SD	14±10	14±8	11±7	16±10	18±15	
Median (IQR)	12 (6, 17)	12 (9, 18)	8 (5, 17)	14 (9, 20)	14 (6, 22)	
Hospital length						0.67
Mean±SD	9±13	7±9	7±11	10±15	14±22	
Median (IQR)	4 (2, 9)	5 (2, 8)	4 (2, 6)	4 (2, 10)	5 (2, 15)	
Operation						0.71
No	59 (69)	19 (68)	12 (60)	21 (75)	7 (78)	
Yes	26 (31)	9 (32)	8 (40)	7 (25)	2 (22)	
Nasal fracture						0.77
No	60 (71)	20 (71)	15 (75)	20 (71)	5 (56)	
Yes	25 (29)	8 (29)	5 (25)	8 (29)	4 (44)	
Bilateral nasal fracture						0.82
No	54 (64)	19 (68)	11 (55)	18 (64)	6 (67)	
Yes	31 (36)	9 (32)	9 (45)	10 (36)	3 (33)	
Mandibular fracture						0.16
No	77 (91)	23 (82)	20 (100)	25 (89)	9 (100)	
Yes	8 (9)	5 (18)	0 (0)	3 (11)	0 (0)	
Bilateral mandibular fracture						0.99
No	83 (98)	27 (96)	20 (100)	27 (96)	9 (100)	
Yes	2 (2)	1 (4)	0 (0)	1 (4)	0 (0)	
Orbital fracture						0.49
No	58 (68)	19 (68)	11 (55)	21 (75)	7 (78)	
Yes	27 (32)	9 (32)	9 (45)	7 (25)	2 (22)	
Bilateral orbital fracture						0.11
No	84 (99)	28 (100)	20 (100)	28 (100)	8 (89)	
Yes	1 (1)	0 (0)	0 (0)	0 (0)	1 (11)	
Maxillary fracture						0.92
No	63 (74)	20 (71)	14 (70)	22 (79)	7 (78)	
Yes	22 (26)	8 (29)	6 (30)	6 (21)	2 (22)	
Bilateral maxillary fractures						0.29
No	79 (93)	27 (96)	19 (95)	26 (93)	7 (78)	
Yes	6 (7)	1 (4)	1 (5)	2 (7)	2 (22)	
Zygomatic fracture						0.43
No	72 (85)	26 (93)	16 (80)	22 (79)	8 (89)	
Yes	13 (15)	2 (7)	4 (20)	6 (21)	1 (11)	
Bilateral zygomatic fractures						0.99
No	84 (99)	27 (96)	20 (100)	28 (100)	9 (100)	
Yes	1 (1)	1 (4)	0 (0)	0 (0)	0 (0)	

Most participants (69%) did not require surgery. The frequency of operations did not significantly differ among the groups (p = 0.71). Similarly, there were no significant differences in specific injuries of nasal (p = 0.77), bilateral nasal fracture (p = 0.82), mandibular (p = 0.16), bilateral mandibular (p = 0.99), orbital (p = 0.49), bilateral orbital (p = 0.11), maxillary (p = 0.92), bilateral maxillary (p = 0.29), zygomatic (p = 0.43), or bilateral zygomatic (p = 0.99) fractures between the groups (Table 1).

The results from the logistic regression model also suggest that there was no statistical difference between safety device utilization and frequency of operation or specific injuries (Table 2). In addition, neither age nor gender was statistically significant with the requirement for surgery (p = 0.20 and p = 0.19, respectively).

**Table 2:** Associations between the safety devices and outcomes CI, confidence interval; REF, reference

Safety devices	Orbital fracture			Bilateral orbital fractures		
	Odds ratio	95% CI	p-value	Odds ratio	95% CI	p-value
Seatbelt and airbag	1	1	REF	1	1	REF
Seatbelt	1.41	0.43, 4.65	0.58	1.73	0.53, 5.65	0.37
Airbag	0.70	0.22, 2.23	0.56	0.70	0.22, 2.26	0.55
None	0.60	0.10, 3.51	0.57	0.60	0.10, 3.51	0.57
Safety devices	Nasal fracture			Bilateral nasal fractures		
	Odds ratio	95% CI	p-value	Odds ratio	95% CI	p-value
Seatbelt and airbag	1	1	REF	1	1	REF
Seatbelt	0.83	0.23, 3.07	0.78	1.73	0.53, 5.65	0.37
Airbag	1.00	0.31, 3.19	1.00	1.17	0.39, 3.55	0.78
None	2.00	0.42, 9.42	0.38	1.06	0.21, 5.21	0.95
Safety devices	Mandibular fracture			Bilateral mandibular fractures		
	Odds ratio	95% CI	p-value	Odds ratio	95% CI	p-value
Seatbelt and airbag	1	1	REF	1	1	REF
Seatbelt	0.18	0, 1.07	0.057	1.40	0, 26.6	0.58
Airbag	0.56	0.08, 3.24	0.705	1.00	0.01, 81.4	1.00
None	0.41	0, 2.51	0.23	3.11	0, 59.11	0.76
Safety devices	Maxillary fracture			Bilateral maxillary fractures		
	Odds ratio	95% CI	p-value	Odds ratio	95% CI	p-value
Seatbelt and airbag	1	1	REF	1	1	REF
Seatbelt	1.07	0.30, 3.78	0.91	1.42	0.08, 24.2	0.81
Airbag	0.68	0.20, 2.31	0.54	2.08	0.18, 24.3	0.56
None	0.71	0.12, 4.20	0.71	7.71	0.61, 97.9	0.12
Safety devices	Zygomatic fracture			Bilateral zygomatic fractures		
	Odds ratio	95% CI	p-value	Odds ratio	95% CI	p-value
Seatbelt and airbag	1	1	REF	1	1	REF
Seatbelt	3.25	0.53, 19.8	0.20	1.40	0, 26.6	0.58
Airbag	3.55	0.65, 19.4	0.14	1.00	0, 19.0	0.50
None	1.63	0.13, 20.4	0.71	3.11	0, 59.1	0.76

## Discussion

Effectiveness of safety devices in preventing facial fractures

This retrospective review contributes significantly to the literature on the relationship between safety device usage and facial fractures in MVC patients, particularly within the challenging context of the Appalachian tri-state area. By integrating our findings with previous research and additional peer-reviewed articles, we can gain deeper insights into the multifaceted nature of MVC-related injuries and the effectiveness of safety interventions.

Cumulative evidence on safety device efficacy

Cummings et al. demonstrated the protective effects of seatbelt usage in reducing facial fractures among MVC patients [10]. Similarly, Haider et al. found that airbag deployment correlated with a lower likelihood of severe facial injuries, highlighting the importance of both seatbelts and airbags in preventing facial trauma [11]. However, our study did not observe a significant difference in the frequency of facial fractures among patients stratified by safety device usage. These disparities may arise from variations in study methodologies, sample populations, or other contextual factors, as discussed by Kent et al. [12].

Considerations for future studies

Moreover, the effectiveness of safety devices in preventing facial fractures may be influenced by factors such as crash severity, vehicle type, and occupant characteristics [14]. Future studies could explore these factors in greater detail, employing larger sample sizes and more diverse study populations to enhance generalizability. In addition, longitudinal studies tracking the long-term outcomes of MVC patients with varying safety device usage could provide valuable insights into the sustained impact of interventions on injury prevention.

Comprehensive injury prevention strategies

Beyond safety devices, educational campaigns and enforcement initiatives are essential components of comprehensive injury prevention strategies. Peek-Asa et al. highlighted the significance of addressing acute traumatic injuries in rural populations, emphasizing the need for targeted interventions tailored to the unique challenges of areas such as the Appalachian tri-state area [15]. In addition, Bowman et al. examined racial disparities in outcomes among traumatic brain injury patients, underscoring the importance of equity in access to trauma care and rehabilitation services [16].

Limitations of the study

While our study adds valuable insights to the literature, several limitations should be acknowledged. The retrospective nature of the review and reliance on medical records may introduce biases and limitations in data collection [17]. Future studies could employ prospective designs or multi-center collaborations to mitigate these limitations and enhance the robustness of findings.

Future directions

Future studies could delve deeper into the mechanisms underlying the relationship between safety device usage and facial fracture outcomes, considering factors such as crash dynamics, vehicle design, and occupant kinematics. In addition, investigations focusing on specific subpopulations, such as elderly drivers or pediatric passengers, could provide targeted insights into injury prevention strategies tailored to vulnerable groups. Moreover, comparative studies across different geographic regions and healthcare settings could elucidate the influence of regional variations in road infrastructure, traffic patterns, and trauma care resources on MVC-related injury outcomes. Such endeavors would contribute to the development of evidence-based guidelines and interventions aimed at reducing the burden of MVC-related injuries and improving patient outcomes across diverse populations.

Expanding the evidence base

Expanding the evidence base through collaborative research efforts and data-sharing initiatives could further enhance our understanding of MVC-related injuries and inform the development of more effective prevention and management strategies. By leveraging advances in data analytics and machine learning techniques, researchers can extract actionable insights from large-scale datasets, facilitating the identification of risk factors, predictive models, and targeted interventions. Furthermore, interdisciplinary collaborations involving researchers, healthcare providers, policymakers, and community stakeholders can foster innovation and drive progress in the field of trauma care and injury prevention. Through sustained efforts and collective engagement, we can address the complex challenges posed by MVCs and work toward creating safer environments for all road users.

## Conclusions

In summary, while previous studies have highlighted the protective role of safety devices such as seatbelts and airbags in mitigating injuries from MVCs, our retrospective review did not find significant associations between safety device utilization and the occurrence of facial fractures in MVC patients within the Appalachian tri-state area. This underscores the need for further research to comprehensively understand the impact of safety interventions on injury outcomes across diverse populations and geographic contexts.

Moving forward, addressing the complex challenges posed by MVC-related injuries requires a holistic approach that extends beyond safety devices alone. By integrating insights from interdisciplinary research efforts and implementing evidence-based interventions, we can work toward creating safer road environments and improving trauma care for individuals affected by MVCs.

## References

[1] (2020). National Highway Traffic Safety Administration (NHTSA): traffic safety facts. https://www.nhtsa.gov/research-data/fatality-analysis-reporting-system-fars.

[2] Centers for Disease Control and Prevention (CDC) (2021). Centers for Disease Control and Prevention (CDC): leading causes of death and injury. Leading Causes of Death and Injury.

[3] World Health Organization (2018). Global status report on road safety. https://www.who.int/publications/i/item/9789241565684.

[4] National Safety Council. (2020) (2022). National Safety Council Injury Facts: the source for injury stats. https://injuryfacts.nsc.org/motor-vehicle/overview/introduction/.

[5] Koppaka R (2011). Ten great public health achievements — United States, 2001-2010. MMWR. Morbidity and Mortality Weekly Report.

[6] Perticone A, Barbani D, Baldanzini N (2024). An enhanced method for evaluating the effectiveness of protective devices for road safety application. Accid Anal Prev.

[7] Hauer E (2000). Safety in geometric design of roads. Transp Res Rec.

[8] Hinchcliff R, Chapman S, Ivers RQ, Senserrick T, Du W (2010). Media framing of graduated licensing policy debates. Accid Anal Prev.

[9] Plasència A, Borrell C (2001). Reducing socioeconomic inequalities in road traffic injuries: time for a policy agenda. J Epidemiol Community Health.

[10] Cummings P, Wells J, Rivara P (2016). Estimating seat belt effectiveness using matched-pair cohort methods. Accid Anal Prev.

[11] Haider AH, Chang DC, Haut ER, Cornwell EE 3rd, Efron DT (2009). Mechanism of injury predicts patient mortality and impairment after blunt trauma. J Surg Res.

[12] Kent R, Henary B, Matsuoka F (2005). On the fatal crash experience of older drivers. Annu Proc Assoc Adv Automot Med.

[13] Young RA (2012). Cell phone use and crash risk: evidence for positive bias. Epidemiology.

[14] Bhalla K, Gleason K (2022). Effects of vehicle safety design on road traffic deaths, injuries, and public health burden in the Latin American region: a modelling study. Lancet.

[15] Peek-Asa C, Zwerling C, Stallones L (2004). Acute traumatic injuries in rural populations. Am J Public Health.

[16] Bowman SM, Martin DP, Sharar SR, Zimmerman FJ (2007). Racial disparities in outcomes of persons with moderate to severe traumatic brain injury. Med Care.

[17] Sadeghi-Bazargani H, Samadirad B, Shahedifar N, Golestani M (2018). Epidemiology of road traffic injury fatalities among car users; a study based on forensic medicine data in East Azerbaijan of Iran. Bull Emerg Trauma.

